# Genes and queens

**DOI:** 10.1186/2041-2223-2-14

**Published:** 2011-06-01

**Authors:** Mark A Jobling

**Affiliations:** 1 Department of Genetics, University of Leicester, University Road, Leicester LE1 7RH, UK

## 

Whether they hate them or they love them, the British certainly are obsessed with their monarchy [1]. In many other nations, the royal family has been dispensed with or relegated to the status of minor celebrities, but the soap opera of the Windsors remains permanently at centre stage here.

The latest instalment has been the marriage of William, son of Charles and Diana, and second in line to the throne, to a 'commoner', Kate Middleton, on 29 April 2011. While not exactly sweeping ashes for a living (the now Duchess is described as a 'former fashion buyer'), this Cinderella has broken new ground. As anyone who has seen the movie *The King's Speech *will know, marrying out of the aristocracy has hardly been embraced with joy in the past. But to those with a genetic or genealogical bent, these royal goings-on are food for thought.

There's a widely held view that royal dynasties are a rather inbred lot, and maybe an injection of genes from a member of the populace would be a good thing: Inbred groups tend to have relatively high frequencies of genetic disorders. Queen Victoria seemed to agree: In a letter to one of her daughters, she wrote, 'I do wish one could find some more black-eyed Princes and Princesses for our children! I can't help thinking what dear Papa said that it was ... when there was some little imperfection in the pure Royal descent that some fresh blood was infused' [2].

The best-known example of a genetic disorder within the British monarchy involves blood more literally: It is haemophilia. Queen Victoria herself was a carrier of this X-linked blood-clotting disorder, which at the time was untreatable in the affected males. Only a few haemophiliacs survived to reproductive age, because any external cut or internal bleeding after a bruise could be fatal. One of Victoria's sons, Leopold, died of it.

While the past few generations of British royals have been haemophilia-free because they descended from Victoria's unaffected son Edward VII, other royal families of Europe have not been so fortunate. Ironically, Victoria's granddaughter (Victoria Eugénie) was brought into the Spanish Bourbon dynasty to revitalise their allegedly degenerate bloodline. The first dramatic evidence of haemophilia was at her baby son Alfonso's circumcision. Queen Victoria's daughter Alice passed the defective gene to her daughter Alexandra, who joined the Russian Romanov dynasty with her marriage to Tsar Nicolas II; their long hoped-for son, Alexis, was a haemophiliac. The imperial family's absorption in their son's sufferings, and their reliance on the monk Rasputin's supernatural help (he apparently stopped the child's internal bleeding by means of a telegram), may have contributed to their own fate. Alexis went on to die in 1918, not of his haemophilia, but as a result of Bolshevik bullets, together with his parents and sisters.

The identity of the remains of the murdered Romanov family was confirmed using genetic methods [3,4]. In this conflicted age of celebrity genomics and privacy concerns, it's interesting to reflect that the first publication, back in 1994 [3], of a DNA sequence from a named living person was that of the current Queen Elizabeth's husband, Prince Philip. His mitochondrial sequence matched that of the remains of the mother in the family group, consistent with the idea that she was indeed his great-aunt, Tsarina Alexandra. More recent analysis of the Romanov bones [5] using next-generation sequencing methods has identified the likely royal haemophilia mutation. It is a substitution at a conserved base within an intron of the X-linked *F9 *gene, predicted to affect RNA splicing and lead to a truncation of the coagulation factor IX, causing haemophilia B, also known as Christmas Disease.

The origin of the royal haemophilia mutation is unknown: it most likely appeared *de novo *in Queen Victoria, although the absence of the disease in her forebears has contributed to the alternative hypothesis that she might have been illegitimate [6]. The mutation may now be extinct, and for concerned living matrilineal descendants in Spain, this could be confirmed by a specific DNA-based test now that its molecular nature is known. The mutation's existence within the British royal family was no signal of inbreeding; in fact, consanguineous marriages among the royals over recent generations have been rare, so Kate Middleton's 'infusion of fresh blood' is probably not going to make much difference.

Kate's recent ancestors and relatives are certainly not very aristocratic, but this says more about the British class system than it does about genetics. Within the past few generations, there have been carpenters, labourers, mechanics and coal miners. The media have been abuzz with 'from Pit to Palace' reports containing interviews with Kate's distant northern cousins Peter Beedle, a chip-shop owner, Judith Purnell, a holistic therapist, and Anna Partington, a hairdresser, about their new royal connections.

The disappointing reality, of course, is that all of us (including William and Kate) are related to each other, albeit in complex ways. A picture of the web of recent shared ancestry is provided by whole-genome SNP analyses; for example, the HapMap project [7] found that any two Europeans (from the CEU sample) shared, on average, 0.34% of their genomes by descent. This is equivalent to the expected mean genome sharing of a pair of third cousins.

The aristocratic families of Britain claim special status through their descent from another William, the Conqueror, born in AD 1027. But in theory, all the indigenous people of Britain have an ancestor in common as recently as about AD 1200, so descent from the conquering William should be no surprise to any of us. The only difference between the aristocracy and the rest is that the former have kept records. Even accounting for the very nonrandom mating habits of our species, a simulation-based study suggests that the most recently living person who is a common ancestor of everyone on the planet lived a mere 3,400 years ago [8]. This paper ends with what is, for *Nature*, a remarkably poetic piece of writing: 'No matter the languages we speak or the colour of our skin, we share ancestors who planted rice on the banks of the Yangtze, who first domesticated horses on the steppes of the Ukraine, who hunted giant sloths in the forests of North and South America, and who laboured to build the Great Pyramid of Khufu' ([8], p.565).

Evocative as these genealogical arguments are, however, they do not correspond to the passage of DNA. With every generation, the amount of DNA inherited from a specific ancestor halves, so if William the Conqueror lived 33 generations ago, we expect our genetic legacy from him, on average, to consist of less than a single base pair of our 3,200-Mbp genome. Not much of a royal inheritance.
